# The Interactive Effects of Transgenically Overexpressed 1Ax1 with Various HMW-GS Combinations on Dough Quality by Introgression of Exogenous Subunits into an Elite Chinese Wheat Variety

**DOI:** 10.1371/journal.pone.0078451

**Published:** 2013-10-22

**Authors:** Xiang Mao, Yin Li, Shasha Zhao, Jian Zhang, Qian Lei, Dandan Meng, Fengyun Ma, Wei Hu, Mingjie Chen, Junli Chang, Yuesheng Wang, Guangxiao Yang, Guangyuan He

**Affiliations:** The Genetic Engineering International Cooperation Base of Chinese Ministry of Science and Technology, Chinese National Center of Plant Gene Research (Wuhan) HUST Part, The Key Laboratory of Molecular Biophysics of Chinese Ministry of Education, College of Life Science and Technology, Huazhong University of Science & Technology (HUST), Wuhan, China; Key Laboratory of Horticultural Plant Biology (MOE), China

## Abstract

Seed storage proteins in wheat endosperm, particularly high-molecular-weight glutenin subunits (HMW-GS), are primary determinants of dough properties, and affect both end-use quality and grain utilization of wheat (*Triticum aestivum* L). In order to investigate the interactive effects between the transgenically overexpressed 1Ax1 subunit with different HMW-GS on dough quality traits, we developed a set of 8 introgression lines (ILs) overexpressing the transgenic HMW-glutenin subunit 1Ax1 by introgression of this transgene from transgenic line B102-1-2/1 into an elite Chinese wheat variety Chuanmai107 (C107), using conventional crossing and backcrossing breeding technique. The donor C107 strain lacks 1Ax1 but contains the HMW-GS pairs 1Dx2+1Dy12 and 1Bx7+1By9. The resultant ILs showed robust and stable expression of 1Ax1 even after five generations of self-pollination, and crossing/backcrossing three times. In addition, overexpression of 1Ax1 was compensated by the endogenous gluten proteins. All ILs exhibited superior agronomic performance when compared to the transgenic parent line, B102-1-2/1. Mixograph results demonstrated that overexpressed 1Ax1 significantly improved dough strength, resistance to extension and over-mixing tolerance, in the targeted wheat cultivar C107. Further, comparisons among the ILs showed the interactive effects of endogenous subunits on dough properties when 1Ax1 was overexpressed: subunit pair 17+18 contributed to increased over-mixing tolerance of the dough; expression of the *Glu-D1* allele maintained an appropriate balance between x-type and y-type subunits and thereby improved dough quality. It is consistent with ILs C4 (HMW-GS are 1, 17+18, 2+12) had the highest gluten index and Zeleny sedimentation value. This study demonstrates that wheat quality could be improved by using transgenic wheat overexpressing HMW-GS and the feasibility of using such transgenic lines in wheat quality breeding programs.

## Introduction

Wheat is one of the most important international crops known for its adaptability to various climatic conditions. Wheat grain is widely used in several flour-based foods primarily due to its unique end-use properties conferred by gluten proteins [1]. Among these proteins, glutenins and gliadins, compose the majority of seed storage proteins and primarily determine the viscoelastic properties of wheat dough. Glutenins are divided into two protein families, high-molecular-weight glutenin subunits (HMW-GS) and low-molecular-weight glutenin subunits (LMW-GS). HMW-GS are major determinants of dough mixing properties and largely determine the end-use quality of wheat [2,3]. In common wheat, the HMW-GS genes are located at the *Glu-A1*, *Glu-B1* and *Glu-D1* loci on chromosomes 1A, 1B and 1D, respectively, with each locus consisting of two tightly linked x-type and y-type alleles [4]. Allelic variations in the HMW-GS composition lead to variations in the structures of glutenin polymers, and therefore affect the rheological properties of dough and wheat end-use quality [5,6]. Particularly, previous studies have correlated stronger dough and better bread-making quality to the HMW-GS subunit 1Ax1 and subunits pair 1Dx5+1Dy10 [2,7,8].

Due to the importance of HMW-GS in determining dough properties, genes of HMW-GS have been used as targets in transgenic wheat to develop cultivars with improved dough functions [9,10,11,12,13]. Specifically, introduction of the subunit 1Ax1 into several common wheat cultivars by genetic transformation and demonstrated the role of transgenic 1Ax1 in enhancing dough quality traits [14,15,16,17,18,19]. Similar results have also been reported in transgenic durum wheat and Tritordeum [20,21,22,23]. However, the effects of 1Ax1 transgene on dough rheological properties varied with different transgenic events. In the weak gluten cultivar L88-31, without 1Ax1 in its genome, expression of exogenous 1Ax1 improved mixing properties [11]. Moreover, such improvement was also observed in the wheat cultivar Bobwhite with good bread-making quality [18]. And greater enhancement in dough quality of wheat also accompanied higher expression levels of 1Ax1 in transgenic lines [24]. However, in the transgenic line, cv Imp, overexpression of 1Ax1 resulted in an overstrong type of dough, similar to those described for lines overexpressing 1Dx5 [25]. Such differential effects of transgenic 1Ax1 largely due to differences in the expression levels of the transgene, interactive effects between transgenic 1Ax1 and endogenous glutenin subunits and the noise of genetic background in different cultivars [24,25,26]. 

Wheat transformation efficiency varies widely among genotypes and is comparatively low. Transformations of most commercial wheat cultivars are not likely to generate high yielding transgenic lines with enhanced agronomic performance, although some commercial wheat cultivars with the positive effects of HMW-GS transgenes on bread-making quality have been successfully generated [14,19,25]. Therefore, an alternate strategy to increase transformation of wheat lines with improved dough properties is by introgression of transgenic HMW-GS subunits from “model” genotypes to advanced cultivars using conventional breeding approaches. Both ILs and NILs can be obtained by conventional crossing/backcrossing, and the resulting strains may carry different alleles of the HMW-GS genes in a similar genetic background. These also serve as ideal genetic stocks to study the interactive effects among different alleles on wheat quality. However, studies thus far have only reported the interactive effects of endogenous alleles at the *Glu-1* loci of non-transgenic wheat cultivars with 1Ax1 subunit expressed normally instead of overexpression. As a result, the interactive and/or additive effects between transgenically overexpressed 1Ax1 and endogenous HMW-GS remain unclear.

To understand the interactions of overexpressed 1Ax1 with endogenous HMW-GS, we selected the B102-1-2/1 transgenic line with stable and robust expression of 1Ax1 to cross with the non-transgenic Chinese elite cultivar, C107, without 1Ax1 in its genome. Previously, we reported introgression of the *1Ax1* transgene into Chinese wheat cultivars Emai 12 and Chuan89-107 [27,28]. Further analyses of dough functionality demonstrated the feasibility of overexpressed 1Ax1 in transgenic lines to improve dough quality in conventional wheat breeding programmes [27]. However, basic studies showing the interactive effects between transgenic and endogenous HMW-GS subunits to improve dough properties are lacking. Therefore, we developed a set of introgression lines (ILs) overexpressing *1Ax1* transgene in similar genetic backgrounds expressing different combinations of endogenous HMW-GS, by crossing B102-1-2/1 with C107. These ILs carrying different HMW-glutenin alleles in a similar genetic background are ideal genetic stocks to evaluate the interactions between transgenic 1Ax1 and endogenous HMW-GS and their effect on wheat quality. The main objectives of the present study were to characterize the storage protein profiles, study the interactive effects between transgenic 1Ax1 and endogenous glutenin subunits, and their impact on the agronomic performance in these ILs. 

## Materials and Methods

### Plant materials

The introgression lines used in this study were generated from a cross between a Chinese commercial variety Chuanmai107 (formerly named as Chuan 89-107 before its release in Sichuan Province, China) and a transgenic wheat line, B102-1-2/1 (paternal line), expressing *1Ax1*. First, the transgenic line, B102-1-2/1, was crossed and backcrossed three times to the recurrent parent C107. Selection of the paternal line B102-1-2/1 from B102-1-2 has been reported previously [27]. Briefly, B102-1-2 is a transgenic line that produced by transformation of wheat line L88-31 with the HMW-GS 1Ax1 under the control of its own endosperm-specific promoter [11]. The maternal line C107 (endogenous HMW-GS represented by 1Bx7+1By9 and 1Dx2+1Dy12) is widely grown in Hubei and Sichuan provinces, China, and is processed into noodles and Chinese steamed bread due to its weak dough strength.

Cross of C107 × B102-1-2/1 was carried out, yielding 15 F_1_ seeds. About 300 F_2_ seeds were analyzed by SDS-PAGE and the composition and expression levels of HMW-GS were determined by gel scanning. F_2_ plants were then backcrossed with C107 three times, generated the backcross progeny BC_3_F_2_ [28]. Lines expressing transgenic 1Ax1 with different combinations of endogenous HMW-GS were analyzed in single half-grains from BC_3_F_2_ seeds by SDS-PAGE. The BC_3_F_2_ plants were self-pollinated and resulted in the BC_2_S_1_ seeds. BC_2_S_1_ plants homozygous for HMW-GS composition were identified by SDS-PAGE of endosperm proteins. Homozygous lines were subsequently self-pollinated for three years and the HMW-GS compositions were re-confirmed by SDS-PAGE. Finally, eight lines (BC_2_S_4_ seeds) expressing transgenic 1Ax1 and one 1Ax1-null control line were selected and used to analyze storage proteins characterization, dough mixing properties and agronomic performance (Table 1).

**Table 1:** Characterization of the storage proteins from introgression lines and their parents^1^.

**Parameters**	**Line**											**LSD _0.05_**	**LSD _0.01_**
	**C107**	**C0**	**C1a**	**C1b**	**C2a**	**C2b**	**C3a**	**C3b**	**C4a**	**C4b**	**B102-1-2/1**		
Transgenic HMW	None	None	1Ax1	1Ax1	1Ax1	1Ax1	1Ax1	1Ax1	1Ax1	1Ax1	1Ax1		
Endogenous HMW	7+9,2+12	7+9, 2+12	7+9, 2+12	7+9, 2+12	7+9	7+9	17+18	17+18	17+18, 2+12	17+18, 2+12	17+18		
Protein content ^2^													
Grain protein content (%)	14.9eD	15.0eD	16.2cB	16.1cB	15.6dC	15.6dC	15.5dC	15.6dC	17.0aA	16.9bA	15.5dC	0.11	0.16
Flour protein content (%)	12.3gF	12.3gF	14.2bB	14.2bB	12.7dD	12.7dD	12.5fE	12.6eE	14.6aA	14.6aA	13.2cC	0.05	0.07
Glutenin (μg mg^-1^ ﬂour) ^3^	2.94gG	2.95gG	3.68cC	3.70cC	3.05fF	3.08fF	3.26dD	3.27dD	4.05bB	4.12aA	3.14eE	0.02	0.03
Gliadin (μg mg^-1^ ﬂour)^3^	2.89gE	2.96fD	3.05eC	3.06deC	3.21aA	3.22aA	3.12cB	3.14bB	3.06deC	3.07dC	3.15bB	0.02	0.03
Glutenin/gliadin	1.02eE	1.00fF	1.21cC	1.21cC	0.95gG	0.96gG	1.04dD	1.04dD	1.32bB	1.34aA	1.00fF	0.01	0.02
HMW composition ^4^													
1Ax1 (% glutenin)	None	None	12.13eE	12.18eE	12.91dD	13.01cC	13.28bB	13.33bB	13.27bB	13.29bB	13.54aA	0.05	0.07
1Bx7 (% glutenin)	9.61aA	9.58aA	8.41bB	8.43bB	9.61aA	9.63aA	None	None	None	None	None	0.04	0.06
1By9 (% glutenin)	5.02aA	4.98bA	4.33cB	4.34cB	5.01abA	4.98bA	None	None	None	None	None	0.03	0.05
1Bx17+1By18^5^ (% glutenin)	None	None	None	None	None	None	14.12bB	14.10bB	12.45cC	12.42cC	14.71aA	0.03	0.04
1Dx2 (% glutenin)	7.98bB	8.07aA	7.09cC	7.03cC	None	None	None	None	5.62dD	5.56dD	None	0.05	0.07
1Dy12 (% glutenin)	8.22aA	8.27aA	6.95bB	6.86cC	None	None	None	None	6.47dD	6.48dD	None	0.04	0.06
x/y ratio ^6^	1.33eE	1.33eE	2.45cC	2.47cC	4.50bB	4.55aA	ND	ND	1.98dD	1.97dD	ND	0.02	0.03
HMW (% glutenin)	30.83cC	30.91cC	38.91aA	38.85aA	27.53eE	27.62eE	27.40eE	27.43eE	37.82bB	37.75bB	28.25dD	0.21	0.28
LMW (% glutenin)	69.17cC	69.09cC	61.09eE	61.15eE	72.47aA	72.38aA	72.60aA	72.57aA	62.18dD	62.25dD	71.75bB	0.22	0.29
HMW/LMW ratio	0.45cC	0.45cC	0.64aA	0.64aA	0.38eE	0.38eE	0.38eE	0.38eE	0.61bB	0.61bB	0.39dD	0.00	0.00

^1^ Values within the same parameter followed by the same letter are not significantly different at 0.05 (small letter) and 0.01 (capital letter) probability level.

^2^ Protein contents were determined by near-infrared reflectance spectroscopy (NIRS) method using an Infratec TM1241 grain analyser and were adjusted to a 14% moisture basis. Average of three replications.

^3^Glutenins and gliadins were determined using the Bradford assay. Average of 10 replications.

^4^ Relative quantities of each HMW-GS were determined by densitometry analysis of SDS-PAGE results and are expressed related to total quantity of the glutenins. Average of 15 replications.

^5^ The relative quantities of the subunit pair 1Bx17+1By18 were analyzed and reported as one parameter due to subunits 1Bx17 and 1By18 could not be clearly separated into two bands on SDS-PAGE gels.

^6^ ND = not determined; Ratios of the x- and y-type HMW-GS for lines C3a, C3b and B102-1-2/1 were not determined as the quantities of 1Bx17 or 1By18 were unable to be densitometerically quantified on SDS-PAGE. To estimate ratios of the x- and y-type HMW-GS for lines C4a and C4b, x/y ratios were calculated as follows:

x/y ratio = (1Ax1 + 1Dx2 + 0.5*(1Bx17+1By18))/(1Dy12 + 0.5*(1Bx17+1By18))

### Field Trials

Eight introgression lines expressing 1Ax1 (C1a, C1b, C2a, C2b, C3a, C3b, C4a, C4b), one 1Ax1-null line (C0) and two parental lines (C107 and B102-1-2/1) were included in the field trials conducted in the years 2011-2012 at the experimental field in the Chinese National Center of Plant Gene Research (Wuhan) HUST Part (Wuhan, Hubei Province, China), using a randomized complete block design with two replicates, as described by Barro et al. [29]. Each plot consisted of six 2.5m long rows with 50 plants per row. The space between rows was 30 cm, and the separation between plots was 50 cm. Plant height, heading date, growth period and number of seeds per spike were determined from 20 plants collected from the two central row of each plot. The 1,000-seed weight and test weight from these lines were measured.

### Seed storage protein characterization

Protein contents in each plots per line were measured on grains and flours harvested during the 2011-12 field trials by near-infrared reflectance spectroscopy (NIRS) method using an Infratec TM1241 Grain Analyzer (Foss North America, Silver Spring, MD) and adjusted to a 14% moisture basis (standard methods recommended by the International Association for Cereal Science and Technology, ICC, no. 159 and no. 202).

Total seed storage proteins were extracted from single kernels obtained from transgenic and parental lines according to Liu et al. [30]. To characterize the storage proteins from each line, gliadins and glutenins were sequentially extracted from 100 mg whole-meal flour according to reference [19]. Gliadins were extracted in 60% (v/v) of aqueous ethanol using a rotary shaker for 1 h. Samples were centrifuged at 13,000 g for 5 min and the supernatant was collected. Glutenins were extracted in 625 mM Tris–HCl pH 6.8, 5% (v/v) 2-mercaptoethanol, 10% (v/v) glycerol, 0.02% (w/v) bromophenol blue and 2% (w/v) dithiothreitol in a 5:1 ratio (ml:mg) to wholemeal and separated using a Tris–borate buffer system and 10% (w/v) acrylamide gels [31]. Gliadin and glutenin contents were determined in the above extracts were determined using a Bradford assay [32]. For densitometry analysis, glutenins from 15 flour samples per line were separated by SDS–PAGE gels and analyzed using a Bio-Rad Quantity One 1-D software version 4.6.2 (Bio-Rad, Hercules, CA). Densitometry method was used in term of its higher reproducibility on storage proteins characterization than HPLC [33].

### Dough testing methods

Seeds from the introgression lines and their parent cultivar C107 harvested during the 2011-12 field trials were used for determining the dough mixing properties. Briefly, after kernel moisture was adjusted to 14% for 24 h at room temperature. 100 g of seeds per line were milled to flour using a Brabender Quandrumat Junior Mill (C.W. Brabender Instruments, Inc., South Hackensack, NJ) following AACC method 26-50 [34]. Samples were mixed to optimum water absorption and the dough mixing properties for each plots per line were determined using a 10 g Mixograph (National Manufacturing Co., Lincoln NE) in three replicates according to the approved AACC method 54-40A [34].

Mixograms and Mixograph parameters were collected from Mixsmart software version 3.8 (AEW Consulting, Lincoln, NE, commercially available through National Manufacturing Division of TMCO, Lincoln NE, USA). In the present study, six major parameters were selected in the present study for they maintain a good representation of dough mixing properties with a minimum of information redundancy [35]. They include two parameters describing the heights of Mixogram curve, midline peak value (MPV) and midline value at 8 min (MTxV)，two describing the widths of curve, midline peak width (MPW) and midline width at 8 min (MTxW). Other parameters were midline peak time (MPT), resistance breakdown (RBD) expressing dough weakening was computed by the ratio of difference of MPV and MTxV with MPV. Results of the mixing parameters from all experiments are given in Table S1. 

Wet gluten content and gluten index, which are measures of dough strength, were determined according to ICC, no. 137/1 and no. 155, using a Perten 2200 Glutomatic System. Zeleny sedimentation value, which relates to the content and quality of gluten, with stronger gluten giving higher values was determined according to ICC no. 137/1.

### Statistical analysis

The experimental data were analyzed using the SPSS 15.0 software. Analysis of variance (ANOVA) and Fisher’s Least Signiﬁcant Difference (LSD) multiple comparisons of means were used to determine significant differences between samples. The statistical significance of pairwise comparisons for mixing parameters was determined using Student’s *t* test. Pearson’s correlation coefﬁcients between protein characterization and dough mixing parameters were also calculated.

### Biosafety Statement

The present study was approved according to the document ‘The Biosafety Permit of Transgenic Plant Research: The Permit for Field Trial of Transgenic Wheat (No. 033)’, authorized by the Ministry of Agriculture of the People’s Republic of China.

## Results

**Figure 1:**
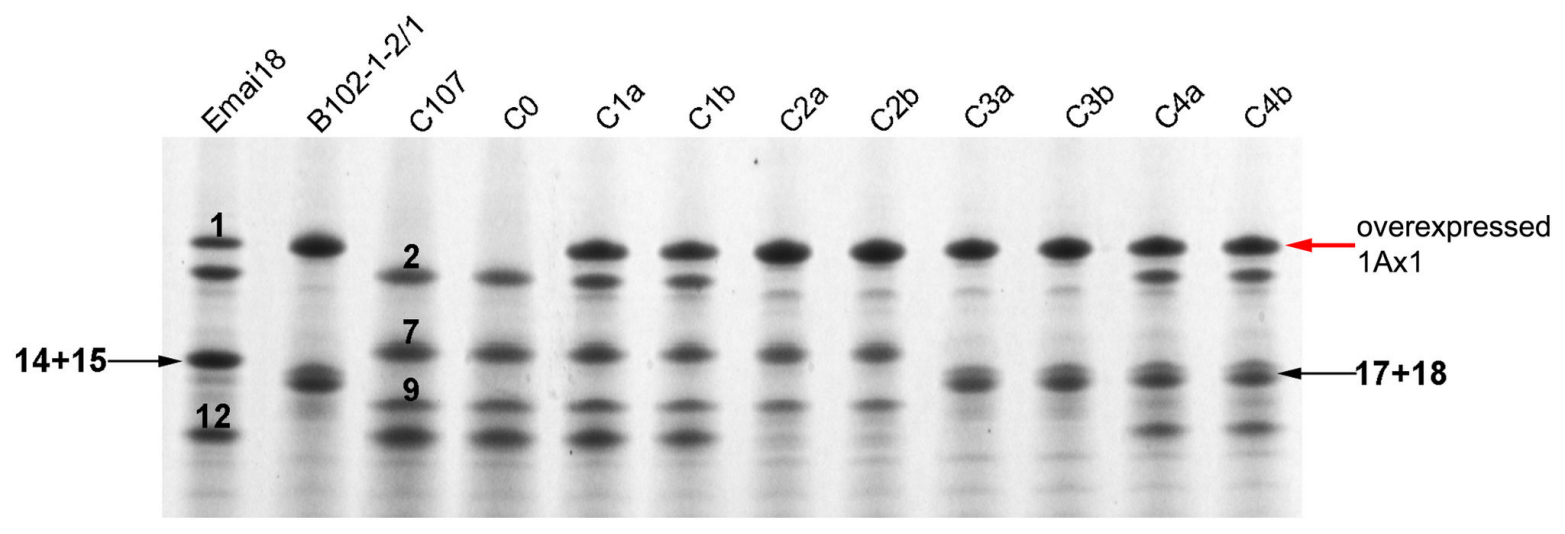
SDS-PAGE patterns of the HMW-GS extracted from seeds of the introgression lines, their parents and wheat variety Emai 18. The endogenous HMW subunits 1Ax1, 1Dx2, 1Bx7, 1By9 and 1Dy12 are indicated by the number 1, 2, 7, 9 and 12, respectively. The endogenous subunit pairs 1Bx14+1By15 and 1Bx17+1By18 are indicated by the number 14+15 and 17+18. The overexpressed 1Ax1 subunits in the transgenic parental line B102-1-2/1 and introgression lines are indicated by red arrow. The wheat variety Emai 18 expressing the endogenous 1Ax1 was used as a control to demonstrate the overexpression of 1Ax1 in the introgression lines. B102-1-2/1 is the paternal line and C107 is the recurrent parent. The introgression lines C1a, C1b C2a, C2b, C3a, C3b, C4a and C4b contains different combinations of endogenous HMW-GS and the transgenically overexpressed 1Ax1, with C0 is a 1Ax1-negative control line.

Eight introgression lines (C1a, C1b, C2a, C2b, C3a, C3b, C4a and C4b) overexpressing the 1Ax1 transgene along with a 1Ax1-negative control C0 and parental lines were subjected to further analyses as described below. The composition of HMW-GS in the parental lines is shown in Figure 1.

### Protein characterization

Total protein contents in the grains (flours) ranged from 14.9% (12.3%) to 17.0% (14.6%) for the recurrent parent cultivar C107 and line C4a introgressed with the *1Ax1* transgene (Table 1). All ILs and transgenic parent line B102-1-2/1 had significantly higher protein contents both in grains and flours than the parental line C107 and the negative control C0. In addition, the glutenin quantities in flour increased from 2.94 μg/mg in C107 to 3.68 and 3.70 in ILs C1a and C1b, respectively. Highest glutenin quantities were detected in lines C4a (4.05) and C4b (4.12). The gliadin fractions increased significantly in all 1Ax1-expressing ILs but only lines C2a (3.21) and C2b (3.22) had higher gliadin contents than B102-1-2/1 (3.15). Interestingly, C1a, C1b, C4a and C4b with higher glutenin contents had relatively lower gliadin contents (Table 1), suggesting a compensation effect in storage protein synthesis and accumulation.

Introgression of the *1Ax1* transgene and endogenous HMW-GS genes encoding 1Bx17+1By18 from B102-1-2/1 to wheat cultivar C107 altered the amounts and proportions of HMW-GS in the endosperms of ILs (Table 1). Transgenic 1Ax1 was overexpressed in all ILs except the negative control, C0 (Table 1 and Figure 1) and the expression levels were significantly lower than the transgenic line B102-1-2/1, which indicated that the high and stable expression of 1Ax1 was preserved after crossing, backcrossing three times and self-pollinating for five generations. Moreover, expression of the transgenic 1Ax1 was associated with a significant decrease in the amounts of endogenous HMW-GS. It is worth noting that such decreases were only observed in the ILs containing both endogenous alleles *Glu-B1* and *Glu-D1* (lines C1a, C1b, C4a and C4b), while those that lacked the *Glu-D1* alleles (lines C2a, C2b, C3a and C3b) had similar levels of endogenous HMW-GS when compared to the two parental lines, C107 and B102-1-2/1, as well as the control C0. Overexpression of 1Ax1 in ILs, significantly increased the ratio of x-type/y-type HMW-GS in comparison with C0 (1.33) and C107 (1.33), varied from 2.45 to 4.55 in lines C1a and C2b. The relative quantities of HMW-GS or LMW-GS in these ILs, also indicated a balance between HMW-GS and LMW-GS due to the different combinations of HMW-GS alleles in the ILs. Lack of the *Glu-D1* alleles in C2a/b (27.53%/27.62%) and C3a/b (27.40%/27.43%) resulted in lower HMW subunit contents than in C1a/b (38.91%/35.85%) and C4a/b (37.82%/37.75%), which contained the *Glu-D1* alleles. However, LMW subunit contents were higher in ILs (C2a/b and C3a/b) without *Glu-D1* alleles than ILs (C1a/b and C4a/b) with the alleles. Further, lower HMW subunit contents in C2a/b (0.38) and C3a/b (0.38) were balanced by an increase in LMW subunits and in turn decreased HMW/LMW ratios when compared to parental line C107 (0.45), C1a/b (0.64) and C4a/b (0.61).

### Dough mixing properties

**Figure 2:**
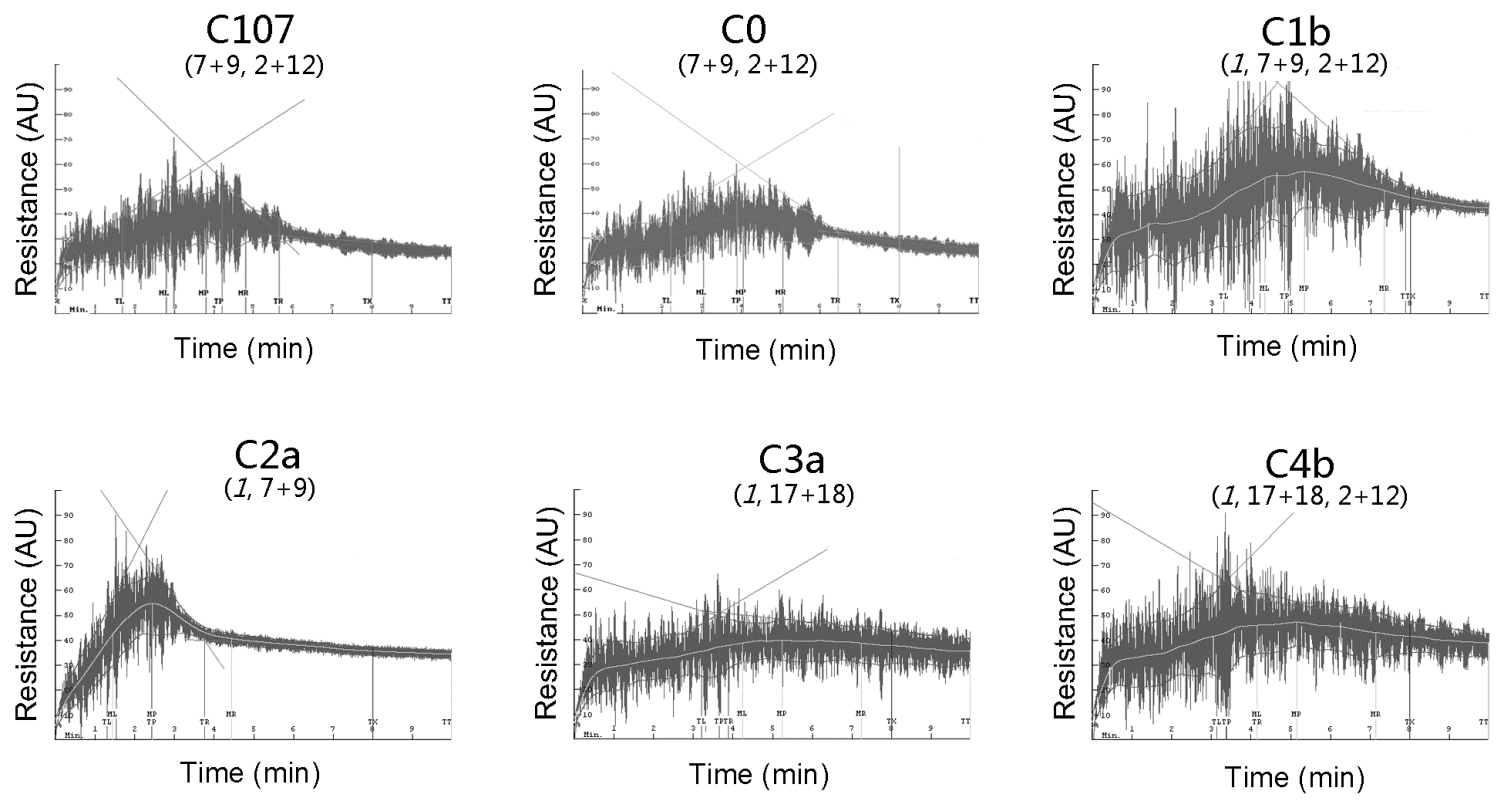
Mixograph curves of dough prepared from introgression lines and their parents. Resistance in arbitrary units (AU) is plotted against time in minutes. As each pairs of introgression lines with the same HMW-GS compositions (lines C1a and C1b, C2a and C2b, C3a and C3b, C4a and C4b) showed similar patterns of Mixograph curves, the Mixograph curves from introgression lines C0, C1b, C2a, C3a and C4b and parental line C107 are shown as representatives. The HMW-GS compositions for each line are indicated in parenthesis, with transgenic 1Ax1 highlighted in Italics.

The potential bread-making quality of the ILs and parental lines were determined using 10 g Mixograph, a widely-used dough testing method. In general, strong doughs have long mixing times, high peak values and band widths and low resistance to breakdown. In Mixograms, stronger doughs have higher and broader mixing curve than weaker doughs. The Mixogram of C0, which is a 1Ax1-null background, showed weak gluten and low resistance to mixing as indicated by a short mixing time and a narrow mixing curve with a large breakdown (Figure 2). A similar curve was observed in the Mixogram of the recurrent parent cultivar C107, which had the same HMW-GS composition (7+9, 2+12) as C0. This demonstrated that the crossing/ backcrossing process during the creation of these ILs hardly had any effect on dough properties.

**Figure 3:**
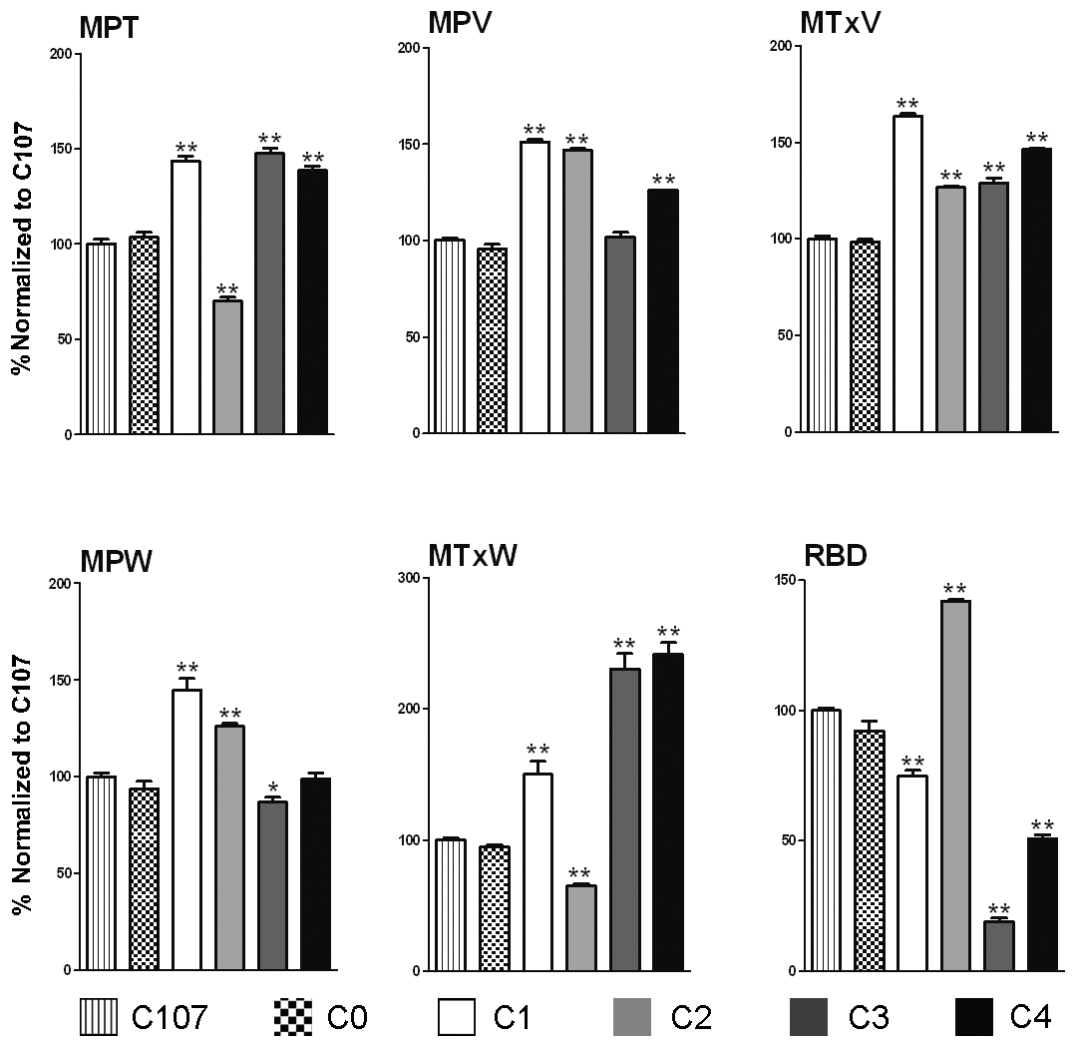
Differential interactive effects of transgenic 1Ax1 and endogenous HMW-GS on dough mixing characteristics. Due to the high similarity of mixing parameter results between lines with the same HMW-GS composition, ILs were subgrouped into four categories, C1 (lines C1a and C1b), C2 (lines C2a and C2b), C3 (lines C3a and C3b) and C4 (lines C4a and C4b), according to the HMW-GS composition of each line. A comparison is made of each mixing parameter among these four subgroups, 1Ax1-null IL C0 and the recurrent parent cultivar C107. All results are expressed normalized to 100% of the results of parental line C107 for each mixing parameter. Complete results are given in Table S1. Data are given as mean ± SEM. *and ** indicates the significant differences within the same mixing parameter at 0.05 or 0.01 probability level, respectively.

Figure 2 shows the Mixograms of ILs overexpressing 1Ax1 (C1b, C2a, C3a and C4b). To more clearly display the effects of overexpressed 1Ax1 on dough property, and to study interactive effects between transgenic 1Ax1 with endogenous HMW-GS, we divided these eight ILs into four subgroups, C1 (HMW-GS are 1, 7+9, 2+12), C2 (1, 7+9), C3 (1, 17+18) and C4 (1, 17+18, 2+12), based on the presence of identical HMW-GS compositions and similar mixing parameters in a pair of ILs (lines C1a and C1b, C2a and C2b, C3a and C3b, C4a and C4b). The complete results of mixing parameters are given in Table S1. In C1, the mixing parameters such as mixing time (MPT), curve values (MPV and MTxV) and curve widths (MPW and MTxW), increased significantly when compared to parent cultivar C107 and negative control CO. On the other hand, large variations in mixing curve patterns and mixing parameter data were observed among the four IL subgroups, demonstrating significant difference in dough properties among the ILs. The highest MPV and relatively high MPT were observed in C1, whereas C2 had the lowest MPT, and C3 had the lowest MPV (Figure 3). It was evident from the Mixograph that the combination of transgenic 1Ax1 with endogenous subunit pairs 7+9 and 2+12 imparted better gluten strength during early mixing than other combinations of transgenic and endogenous HMW-GS. Moreover, the mixing parameter data for C4 showed that the overexpression 1Ax1 along with endogenous HMW-GS 17+18 and 2+12 gave better overall mixing properties (gluten strength, resistance to extension and over-mixing tolerance) than other HMW-GS combinations.

**Figure 4:**
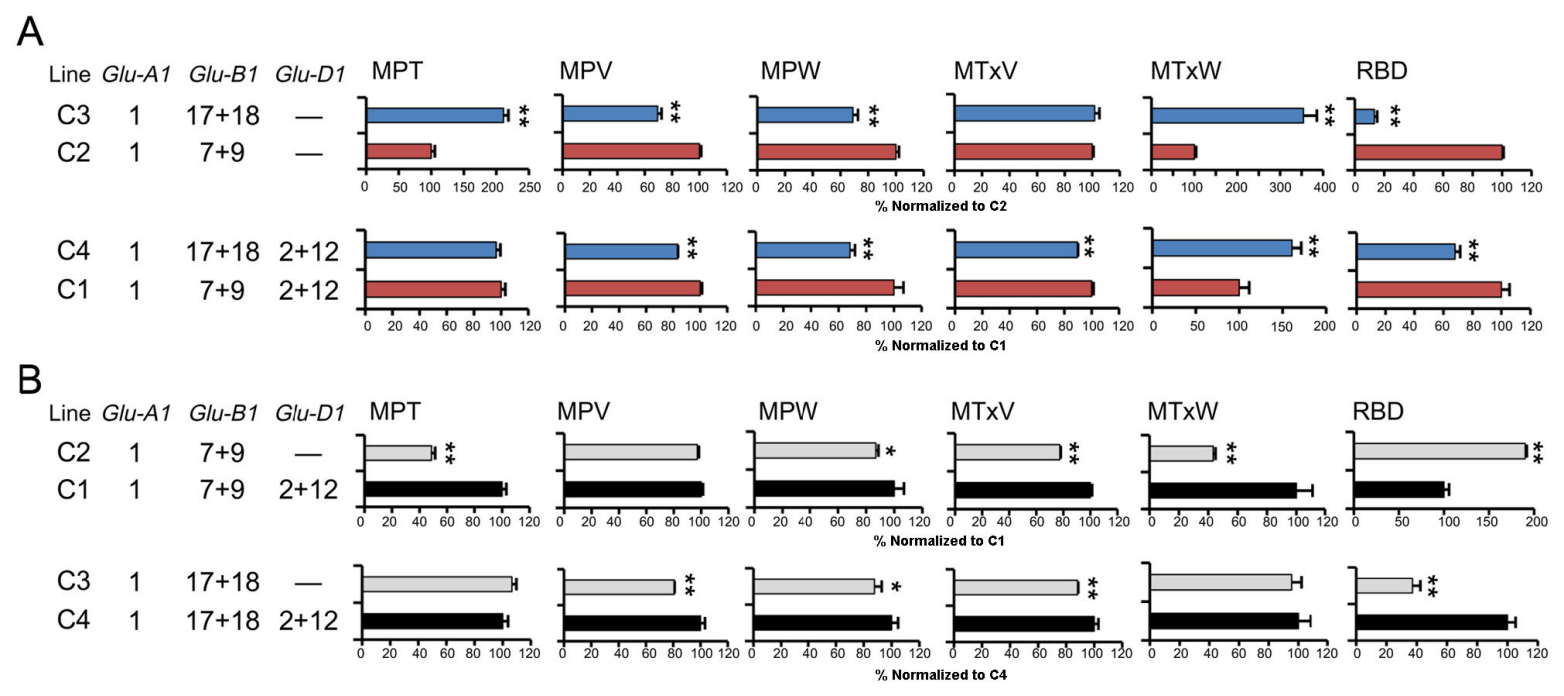
Effects of subunit pair 7+9, 17+18 (A) or 2+12 (B) on dough mixing characteristics in the presence of transgenic 1Ax1. A. To study the effects of subunit pair 7+9 or 17+18 on dough property, mixing parameter results for lines C3a and C3b were compared with lines C2a and C2b, expressed normalized to 100% C2 results, and parameters for lines C4a and C4b were compared with lines C1a and C1b, expressed normalized to 100% of C1 results. The results for lines containing subunit pair 17+18 (C3a, C3b, C4a and C4b) are indicated in blue bar and results for lines containing subunit pair 7+9 (C1a, C1b, C2a and C2b) indicated in red bar. B. To study the effects of subunit pair 2+12 on dough property, mixing parameter results for lines C2a and C2b were compared with lines C1a and C1b, expressed normalized to 100% C1 results, and parameters for lines C3a and C3b were compared with lines C4a and C4b, expressed normalized to 100% of C4 results. The results for lines containing subunit pair 2+12 (C1a, C1b, C4a and C4b) are indicated in grey bar and results for lines without subunit pair 2+12 (C2a, C2b, C3a and C3b) indicated in black bar. Complete results are given in Table S1. Data are given as mean ± SEM. *and ** indicates the significant differences within the same mixing parameter at 0.05 or 0.01 probability level, respectively.

The impact of subunit pairs 17+18, 7+9 or 2+12 on dough mixing properties in combination with transgenic 1Ax1 is shown in Figure 4. These results show that replacement of subunit pair 17+18 with 7+9 significantly increased MPV and MPW during early mixing but also decreased MTxW and RBD, suggesting an enhancement in dough over-mixing tolerance by 17+18 (Figure 4A). In contrast, addition of subunits 2+12 affected the mixing parameters differently. For example, a comparison of the mixing parameters between the C2 and C1 showed significant increase MPT, MPW, MTxV and MTxW, but a drastic decrease in RBD. Between C3 and C4, addition of the subunit pair 2+12 resulted in a dramatic increase in RBD (Figure 4B). These differences probably reflect differences in the ratios of x-type/y-type HMW-GS and HMW: LMW subunits, which were altered only by the addition of the subunit pair 2+12.

**Figure 5:**
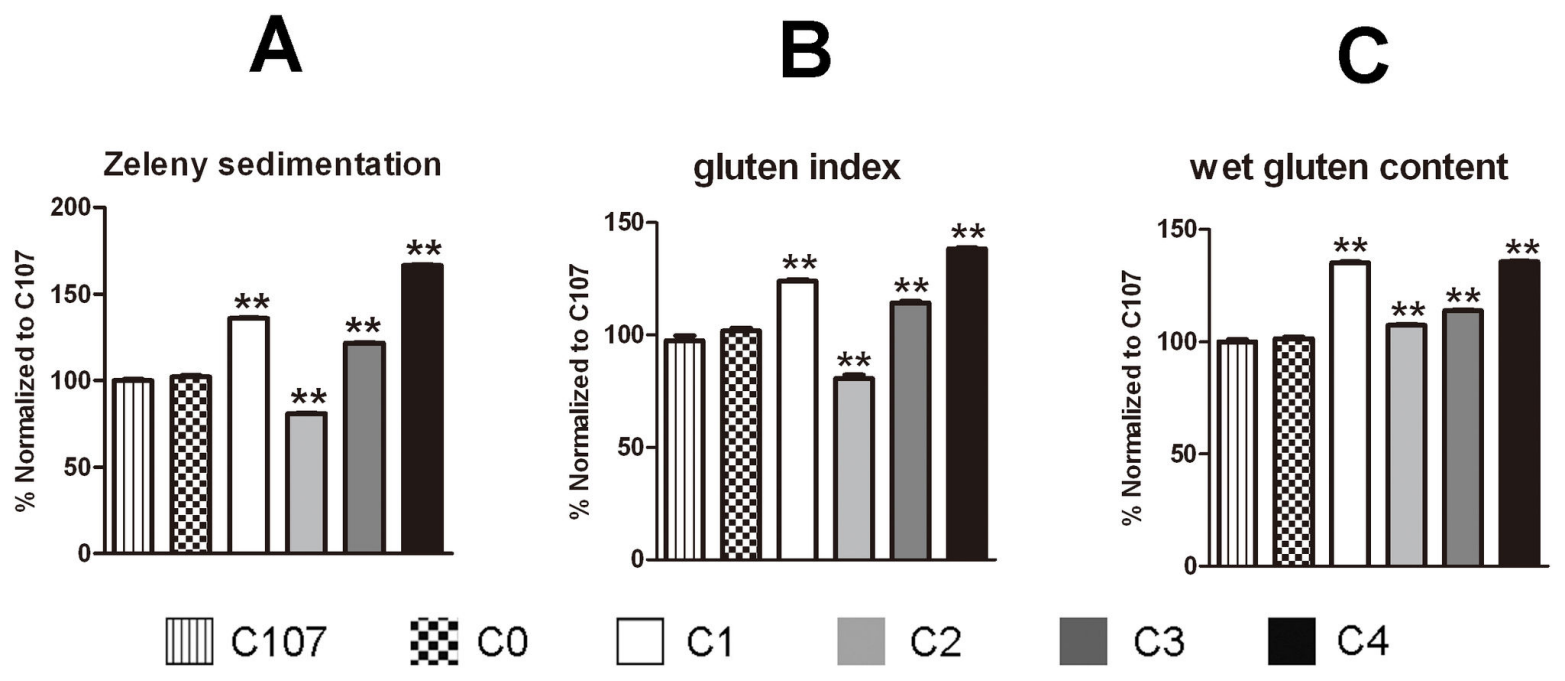
Comparison of other parameters associated with dough quality for the introgression lines and their parents. Due to the high similarity of parameter results associated with dough quality between lines with the same HMW-GS composition, ILs were subgrouped into four categories, C1 (lines C1a and C1b), C2 (lines C2a and C2b), C3 (lines C3a and C3b) and C4 (lines C4a and C4b), according to the HMW-GS composition of each line. A comparison is made of each parameter among these four subgroups, 1Ax1-null IL C0 and the recurrent parent cultivar C107. All results are expressed normalized to 100% of the results of parental line C107 for each parameter. Complete results are given in Table S1. Data are given as mean ± SEM. *and ** indicates the significant differences within the same mixing parameter at 0.05 or 0.01 probability level, respectively. A. Comparison of Zeleny sedimentation value; B. Comparison of gluten index; C. Comparison of wet gluten content.

The Zeleny sedimentation values were not significantly different between the parental line C107 and the negative control line C0, however, significant differences were observed between each IL and C107 (Table S1). We also divided these eight ILs into four subgroups as described above, as shown in Figure 5A, ILs C2 showed significantly lower Zeleny sedimentation values than C107 while C1, C3 and C4 showed higher values. Further, ILs C4 had the greatest increase in Zeleny sedimentation value.

A similar pattern was observed in gluten index test (Figure 5B). While ILs C4 had he highest gluten index (ca. 96), C2 had the lowest (ca. 56 in Table S1). Notably, gluten index increased about 28 and the Zeleny sedimentation value about 20 ml.

Results of wet gluten content were not consistent with Zeleny sedimentation values and gluten index. Each IL had significantly higher wet gluten content than C107, while ILs C2 and C4 had similar wet gluten content (Figure 5C).

### Agronomic performance

It has been reported that transgenic wheat had the relatively poorer agronomic characteristics than their control lines [22,29,36,37]. To address whether the expression of transgenic 1Ax1 affected agronomic performance, all the agronomic traits were analyzed in the ILs and parental lines (Table 2). Statistical differences were observed in almost the agronomic traits among all field-grown lines, but the eight ILs showed similar growth periods (ca. 192 days). Despite these differences between ILs and parental lines, similarities were also observed: all ILs exhibited agronomic performance similar to their recurrent parent C107, but the grain yield-relating characters for all ILs (No. of seeds per spike, 1000-seed weight and test weight) were significantly higher than the transgenic line, B102-1-2/1.

**Table 2:** Agronomic performance of introgression lines and their parents^a^.

**Trait**	**Line**											**LSD_0.05_**	**LSD _0.01_**
	**C107**	**C0**	**C1a**	**C1b**	**C2a**	**C2b**	**C3a**	**C3b**	**C4a**	**C4b**	**B102-1-2**		
Heading date (days)	140.2deDE	140.9cC	141.1cC	140.9cC	141.9bB	141.2cC	141.1cC	140.5dD	140.1eE	140.9cC	153.1aA	0.26	0.34
Growth period (days)	191.7cC	191.9cC	192.3bcBC	191.9cC	193.1bB	192.4bcBC	191.6cC	191.7cC	191.7cC	192.3bcBC	206.2aA	0.77	1.01
Plant height (cm)	88.6fF	89.9dD	89.1eE	88.9efEF	91.3bB	89.2eE	89.1e E	90.6cC	90.1dD	88.9efEF	104.1aA	0.26	0.34
No. of seeds per spike	42.6bB	42.4bcB	42.3bcB	42.2cBC	41.7dC	42.1cBC	42.3bcB	42.4bcB	43.2aA	42.4bcB	28.3eD	0.41	0.54
1000-seed weight (g)	44.7bB	44.3cC	44.6bcBC	44.5bcBC	41.9eE	44.3cC	44.5bcBC	43.2dD	45.2aA	44.6bcBC	30.1fF	0.21	0.27
Test weight (g/L)	791bB	781dD	788cC	788cC	756fF	786cC	787cC	766eE	796aA	788cC	723gG	1.82	2.40

^a^ Values within the same parameter followed by the same letter are not significantly different at 0.05 (small letter) and 0.01 (capital letter) probability level.

## Discussion

### The introgression lines with various combinations of transgenic 1Ax1 and endogenous HMW-GS are useful tools to evaluate allelic effects on grain quality

The influences of HMW-GS on dough rheological properties and grain quality in wheat were studied previously by using recombinant inbred lines [38], near isogenic lines [39] and wheat germplasm generated by breeding programs [40]. Most of these studies reported only the interactive effects of endogenous alleles at *Glu-1* loci. Previous studies on transgenic wheat lines overexpressing HMW-GS have demonstrated that transformation with the gene encoding 1Ax1 can result in significant effects on the compositions of gluten protein and dough functional properties, including both positive effects and negative effects [19,25]. The differential effects of transgenic 1Ax1 largely resulted from variations in expression levels, interactive effects between transgenic 1Ax1 and endogenous glutenin subunits and the noise of genetic background in different cultivars. Different transgenic wheat lines were shown to have different levels of 1Ax1 expression, which could depend on many factors, such as copy numbers and site of gene insertion. Generally, overexpression of 1Ax1 were found in most transgenic wheat lines while non-transgenic wheat cultivar only contained a normal expression of 1Ax1, however, previous studies do not clearly reveal the interactive and/or additive effects between transgenically overexpressed 1Ax1 and endogenous HMW-GS. Furthermore, several studies have shown that introgression of HMW-GS transgenes from “model” to commercial genotypes is a valid strategy to fully utilize the HMW-GS transgenes and to increase grain quality in commercial cultivars [27,41]. Therefore, the study of the interactive effects between transgenically overexpressed 1Ax1 and endogenous HMW-GS on dough quality by introgression of transgenes from “model” to commercial genotypes is necessary and important. In addition, NILs and ILs have proven to be efficient genetic stocks for accurately dissecting the additive and/or interactive effects of individual HMW subunits on wheat grain quality [26,39,42]. In the present study, we developed 8 ILs with overexpressing 1Ax1, as well as one IL without the *1Ax1* transgene, and elucidated the effects of overexpressed 1Ax1 overexpression on gluten protein composition and dough mixing properties. We also investigated interactive effects of three endogenous HMW-GS pairs (7+9, 17+18, 2+12) with the overexpressed 1Ax1 on dough functionality. Our results revealed that in these ILs the agronomic traits were similar to the commercial variety C107 rather than the transgenic line B102-1-2/1. This demonstrated that improvement of dough properties can be achieved in conventional breeding programs by using transgenic wheat lines expressing HMW-GS without negative effects on yield or other important agronomic traits. Meanwhile, we also confirmed that the high level and stable expressions of 1Ax1 were preserved after crossing, backcrossing three times and five generations of self-pollination, these processes have lasted at least ten years. The proven stability of transgenically overexpressed 1Ax1 in ILs related to the application value of all transgenically overexpressed HMW-GS on improving wheat dough quality.

### Over-expression of transgenic 1Ax1 is compensated by endogenous gluten proteins

Compensatory effects of wheat storage proteins have been reported previously by several groups in transgenic lines where transgenic HMW-GS were overexpressed by genetic transformation or gliadins were down-regulated by RNAi technology [19,23,25,43]. In transgenic lines B102-1-2 overexpressing 1Ax1, no compensatory effect was observed in endogenous HMW-GS (subunit pair 17+18), which is likely due to the lack of *Glu-D1* allele [33]. We observed similar storage protein profiles in lines C3a, C3b and B102-1-2/1 although a slight decrease in HMW-GS was observed in ILs C3a and C3b (Table 1), which could be due to the lower flour protein contents in these ILs than B102-1-1/1. In field grown transgenic lines from commercial wheat cultivars overexpression of 1Ax1 was compensated by endogenous HMW-GS and LMW-GS [25]. In transgenic lines of durum wheat with expression of the *1Ax1* transgene, significant decrease in Bx- and By-types HMW-GS were associated with the expression of HMW-GS transgene, and the levels of LMW-GS were lower than in control lines [23]. In the present study, overexpressed 1Ax1 was only compensated by LMW-GS and gliadins in ILs containing the null allele at the *Glu-D1* locus (ILs C2 and C3), while in ILs expressing the subunit pair 2+12 (ILs C1 and C4) an overall decrease in endogenous HMW-GS and LMW-GS, with a relative shift in gliadin contents was detected. Our results along with previous data suggest that a balance between endogenous storage proteins and transgenically expressed HMW-GS is critical in wheat lines containing active alleles at both *Glu-B1* and *Glu-D1* loci. Further, endogenous HMW and LMW subunits tend to decrease in response to overexpression of the HMW-GS transgenes. However, the molecular mechanism regulating this compensatory phenomenon remains unclear. On one hand, compensation between storage proteins probably reflect the competition for amino acids during protein synthesis and accumulation, which is supported by several lines of evidence in both wheat and maize. Down-regulation of γ-gliadin was balanced by increase in the ω- and α-gliadins in transgenic wheat lines and *in silico* amino acid composition analysis showed no difference in the γ-gliadins silenced lines [43]. Similarly, another group demonstrated that RNAi-induced silencing of α-zein in maize led to complementary increase of non-zein storage proteins and can rebalance the nitrogen sink of maize seeds [44]. The sulfur sink in maize seeds revealed the complexity of amino acid competition and its influence on balancing between groups of storage proteins: overexpression of the methionine-rich proteins was associated with reduction in cysteine-rich storage proteins and this could be at the translational level [45]. On the other hand, previous studies have demonstrated that expression of wheat prolamin genes is controlled in a specific spatial-temporal pattern by several transcriptional factors (TFs), with two major TFs known as storage protein activator (SPA) and prolamin-box binding factor (PBF) [46]. *In silico* analysis on the promoters of LMW-GS genes provided further evidence that transcription regulation played an important role in coordinating the expression levels between different LMW-GS subgroups [47]. Therefore, it appears that the balance between groups of storage proteins in wheat is possibly the result of integrated control of prolamin gene expression, protein synthesis and amino acid metabolism flux at different levels. These ILs created in this study may be useful genetic stocks to study the mechanism underlying the compensatory phenomenon of storage proteins in wheat.

Although HMW-GS are the key determinants of dough mixing properties, LMW-GS and gliadins were also found to influence the bread-making quality of wheat. While glutenins play a major role in determining dough visco-elasticity, gliadins contribute by inducing minor or modifying effects. Altering the glutenin-to-gliadin ratio has a complex effect on the elongational properties of doughs [48,49], reflecting the different rheological properties of glutenin and gliadin. Further, allelic variations in LMW-GS were found to be related to different dough properties in wheat, particularly, LMW-GS fractions were significantly correlated with dough resistance [50,51,52]. 

In our study, indeed, the introgression of transgenically overexpressed 1Ax1 led to variations in the contents and compositions of LMW-GS and gliadins, which may have further influenced the dough quality. However, a strong negative correlation (**) was observed only between LMW content and MTxV value in the ILs created in this study. Interestingly, MTxV was not a key parameter when compared to MPT, MTxW, MPV or RBD. It is likely that identical backgrounds in all ILs after three backcrosses with the recurrent parent, C107, could have resulted in only a few differences in compositions of both LMW-GS and gliadins, which may have limited their effects on dough properties.

### Effects of overexpressed 1Ax1 and endogenous HMW-GS on dough properties

Dough mixing and developments are of great importance in processes for making flour-based foods. Due to the determinant role of HMW-GS in dough rheological properties, the effects of individual HMW-GS on dough properties and end-use qualities of wheat have been extensively studied on transgenic lines of wheat expressing/overexpressing of individual subunits [10,18,11,16,17,19]. Generating these transgenic lines allows determination of the impacts of individual subunits on different rheological properties. Such studies have demonstrated that expression of subunit 1Ax1 or the combination of 1Dx5 and 1Dy10 improves not only dough strength but also dough stability and over-mixing tolerance [19,53]. However, high-level expression of subunit 1Ax1 or 1Dx5, had negative effects on dough strength, stability or resistance to extension [16,17,19,25,54,55,56]. In addition, the behavior of individual transgenic subunits was also affected by endogenous HMW-GS compositions. In a transgenic line expressing the *1Ax1* transgene and containing the endogenous HMW-GS pairs 7*+8 and 2+12, expression of the foreign subunit resulted in increased dough strength and mixing tolerance but had little effect on dough extensibility [19]. In another study, expression of transgenic 1Ax1 in the cultivar, Canon (HMW-GS compositions are 2*, 7+9, 2+12), increased all aspects of dough properties, including gluten strength, dough extensibility and resistance to extension; while 1Ax1 expression in another cultivar, Cadenza (HMW-GS compositions are 1, 14+15, 5+10), only improved dough stability and extensibility [25].

In our study, overexpression of 1Ax1 in the cultivar, C107 (HMW-GS compositions are 7+9, 2+12), improved the overall dough mixing performance, with significant increases in all six mixing parameters, Zeleny sedimentation value, wet gluten content and gluten index (Figure 3 and Figure 5). Our results along with the previously reported data indicate that overexpression of transgenic 1Ax1 may possibly increase dough strength without negative effects in *Glu-A1*-null genetic backgrounds where *Glu-B1* and *Glu-D1* alleles do not contribute to good bread-making quality. Comparison of the introgression lines with various HMW-GS combinations showed that the combination of overexpressed 1Ax1 with the and endogenous subunit pairs 17+18 and 2+12 resulted in the greatest increase in quality, followed by the combination of 1Ax1 and endogenous subunit pairs 7+9 and 2+12 with the same wet gluten content in both (Table 1, Figure 2 and Figure 3). In contrast, combinations of transgenic 1Ax1 with HMW-GS pair 7+9 or 17+18 resulted in poor dough properties, demonstrating that an appropriate balance of x-type and y-type subunits is required. This was further supported by significant correlation coefficients between the ratio of x-type/y-type subunits and dough mixing characteristics (Table S2). Notably, ILs C2 showed the poorest dough properties, even poorer than recurrent parent C107, although C2 had higher wet gluten content (Figure 5B). Such negative correlation between wet gluten content and gluten index, and Zeleny sedimentation value has been reported previously. Recently, gluten index was reported as a better measure of wheat processing quality than wet gluten content. It appears to have a positive correlation with major parameters such as Farinograph, Extensograph and Zeleny sedimentation value. And Zeleny sedimentation value was a good parameter useful in balancing wheat gluten quality and quantity. 

Comparison of the ILs null for *Glu-D1* (C2a, C2b C3a and C3b) showed that although both C2 and C3 had poor dough qualities, they differed in dough mixing performance with C2 showing significantly lower dough strength and over-mixing tolerance but C3 only exhibiting poor dough property in the early stage of dough development (Figure 2 and Figure 3). In addition, ILs C3 had higher Zeleny sedimentation value and gluten index than C2, indicating that C3 had better dough quality. The differential dough performance between C2 and C3 could be likely due to differences in protein characteristics between subunit pairs 17+18 and 7+9.

Comparison of the various HMW-GS compositions in these ILs not only showed the effects of overexpressed 1Ax1 but also revealed interactive effects of endogenous HMW-GS on dough property in the presence of overexpressed 1Ax1. Comparison of the ILs C4 with C1, or the ILs C3 with C2 showed similar trends in dough mixing parameters, indicating that subunit pair 7+9 imparts better dough strength and extensibility during early dough mixing, while subunit pair 17+18 confers excellent dough stability and over-mixing tolerance (Figure 4A). Further analysis of the dough mixing data (Figure 4B) showed that addition of the subunit pair 2+12 had differential effects on over-mixing tolerance. The combination of 2+12 with overexpressed 1Ax1 and endogenous subunit pair 7+9 improved all mixing parameters analyzed in this study compared to C2; whereas the addition of 2+12 on overexpressed 1Ax1 and subunit pair 17+18 significantly decreased the over-mixing tolerance of dough. There are two likely reasons to explain our findings, one is the protein characteristics of glutenin subunits encoded by the 1Bx7 and 1By9 genes and second is the increased grain protein contents by the addition of 2+12 that indirectly affected mixing characteristics. Our data also support these two explanations: on one hand, both grain and flour protein contents in ILs C4 were highest among all ILs; on the other hand, the correlation analyses showed that the subunit pair 2+12 negatively correlated with the ratio of x-type/y-type of HMW-GS which is also associated with resistance to breakdown (Table S2). A caveat of this study is that the differential effects of endogenous HMW-GS on dough property reported here should be interpreted with consideration of the presence of 1Ax1 overexpression. Nevertheless, these findings should be of particular value in utilizing transgenically overexpressed HMW-GS subunits in conventional breeding programs with the aim of end-use quality improvement.

## Conclusions

In summary, we report the production, characterization and dough properties of 8 introgression lines overexpressing the *1Ax1* transgene, which could be beneficial for making a range of flour-based foods. Our results demonstrated that transgenic overexpression of 1Ax1 can be introgressed into commercial wheat cultivars by conventional crossing and could improve wheat grain quality in the genotype of cultivar C107 which is poor in dough quality and null for the *Glu-A1* locus, improved the overall dough mixing performance. In particular, gluten strength and resistance to extension during early mixing were significantly increased. Comparison of these ILs revealed the differential effects of individual endogenous subunits on dough quality: in combination with transgenic 1Ax1, subunit pair 17+18 gave better dough quality than subunit pair 7+9, which had greater over-mixing tolerance of dough. The *Glu-D1* allele was required for good dough quality because it provided an appropriate balance between the ratio of x-type/y-type subunits and increased protein content in the grain. Moreover, agronomic performance of these ILs demonstrated that conventional crossing method is a valid strategy to create transgenic lines overexpressing HMW-GS without loss of any superior agronomic traits in commercial cultivars. Lastly, the effects of overexpressed 1Ax1 and its combinations with the various HMW-GS compositions on dough quality will be important and useful for fully utilizing the transgenic lines of wheat expressing the HMW-GS transgenes and for wheat quality breeding.

## Supporting Information

Table S1 **Parameters associated with dough quality in the introgression lines and their parental lines.**
(DOC)

Table S2 **Correlations among HMW-GS subunits, protein characteristics and Mixograph parameters in the introgression lines and their parents.**
(DOC)
