# Supplementary material for: The Interactive Effects of Transgenically Overexpressed 1Ax1 with Various HMW-GS Combinations on Dough Quality by Introgression of Exogenous Subunits into an Elite Chinese Wheat Variety
Source: PLoS One. 2013 Oct 22;8(10):e78451. doi: 10.1371/journal.pone.0078451 (PMC3805546; doi:10.1371/journal.pone.0078451)
Supplement: Table S1 — Parameters associated with dough quality in the introgression lines and their parental lines. (DOC) [file pone.0078451.s001.doc]

**Table S1.** Parameters associated with dough quality in the introgression lines and their parental lines ^a,b^.

| **Parameters** | **Line** | | | | | | | | |  | **LSD _0.05_** | **LSD _0.01_** |
| --- | --- | --- | --- | --- | --- | --- | --- | --- | --- | --- | --- | --- |
|  | **C107** | **C0** | **C1a** | **C1b** | **C2a** | **C2b** | **C3a** | **C3b** | **C4a** | **C4b** |  |  |
| Transgenic HMW | None | None | 1Ax1 | 1Ax1 | 1Ax1 | 1Ax1 | 1Ax1 | 1Ax1 | 1Ax1 | 1Ax1 |  | |
| Endogenous HMW | 2+12,7+9 | 2+12,7+9 | 2+12,7+9 | 2+12,7+9 | 7+9 | 7+9 | 17+18 | 17+18 | 2+12,17+18 | 2+12,17+18 |  | |
| Zeleny sedimentation value (ml) | 30.4fD | 31.0eD | 41.2cB | 41.3cB | 24.4gE | 24.8gE | 37.0dC | 36.9dC | 50.3bA | 50.8aA | 0.5 | 0.69 |
| Gluten index | 68.95dD | 70.92dD | 86.42bB | 86.25bB | 57.25eE | 55.01eE | 79.15cC | 80.05cC | 96.02aA | 96.79aA | 2.62 | 3.57 |
| Wet gluten content (%) | 26.83dD | 27.17dD | 36.27aA | 36.20aA | 28.73cC | 28.77cC | 30.33bB | 30.67bB | 36.23aA | 36.43aA | 0.53 | 0.72 |
|  | | | | | | | | | | | | |
| Mixograph |  | | | | | | | | | | | |
| Midline peak time (min) | 3.63dD | 3.76dD | 5.32abAB | 5.12 bcBC | 2.64eE | 2.44eE | 5.23abABC | 5.49aA | 5.19bABC | 4.87cC | 0.26 | 0.36 |
| Midline peak value (% Torque) | 37.49eE | 35.93fF | 57.98aA | 55.36bB | 55.34bB | 54.76bB | 38.83dD | 37.59eDE | 46.98cC | 47.56cC | 0.97 | 1.33 |
|
| Midline peak width (% Torque) | 18.73dDE | 17.56deDE | 25.97bAB | 28.35aA | 22.93cC | 24.23bcBC | 15.98eE | 16.54 eDE | 19.13dD | 17.97deDE | 2.06 | 2.82 |
|
| Midline value at 8 min (% Torque) | 28.23fE | 27.76fE | 46.31aA | 46.13aA | 36.09deCD | 35.47eD | 36.88dC | 35.94 eCD | 40.95cB | 41.77bB | 0.81 | 1.11 |
|
| Midline width at 8 min (% Torque) | 5.30eD | 5.01eD | 8.29cC | 7.65dC | 3.56fE | 3.34fE | 12.44bAB | 12.02bB | 12.40bAB | 13.22aA | 0.62 | 0.84 |
|
| Resistance  breakdown (%) | 24.70bB | 22.74cC | 20.13dD | 16.67eE | 34.78aA | 35.23aA | 5.02gG | 4.39gG | 12.84 fF | 12.17fF | 1.27 | 1.73 |

^a^ Values within the same parameter followed by the same letter are not significantly different at 0.05 (small letter) and 0.01 (capital letter) probability level

^b^ Values are the average of three replications
