# Supplementary material for: The Interactive Effects of Transgenically Overexpressed 1Ax1 with Various HMW-GS Combinations on Dough Quality by Introgression of Exogenous Subunits into an Elite Chinese Wheat Variety
Source: PLoS One. 2013 Oct 22;8(10):e78451. doi: 10.1371/journal.pone.0078451 (PMC3805546; doi:10.1371/journal.pone.0078451)
Supplement: Table S2 — Correlations among HMW-GS subunits, protein characteristics and Mixograph parameters in the introgression lines and their parents. (DOC) [file pone.0078451.s002.doc]

**Table S2.** Correlations among HMW-GS subunits, protein characteristics and Mixograph parameters in the introgression lines and their parents

|  | **Transgenic 1Ax1** | **17+18** | **7+9** | **2+12** | **glu/glia** | **HMW/LMW** | **x/y** | **LMW** | **glia** |
| --- | --- | --- | --- | --- | --- | --- | --- | --- | --- |
| GPC | 0.74** | 0.51* | -0.60** | -0.08ns | ND | ND | ND | ND | ND |
| FPC | 0.60** | 0.30ns | -0.42ns | 0.18ns | ND | ND | ND | ND | ND |
| glu/glia | 0.43ns | 0.44ns | -0.55* | 0.32 | ND | ND | ND | ND | ND |
| HMW/LMW | 0.21ns | 0.019ns | -0.14ns | 0.58* | 0.89** | ND | ND | ND | ND |
| x/y | 0.85** | ND | -0.01ns | -0.98** | -0.21ns | -0.27ns | ND | ND | ND |
| MPT | 0.38ns | 0.65** | -0.70** | 0.10ns | 0.69** | 0.55* | -0.47ns | -0.53* | -0.05ns |
| MPV | 0.66** | -0.22ns | 0.14ns | -0.24ns | 0.34ns | 0.44ns | 0.79** | -0.43ns | 0.58* |
| MPW | 0.34ns | -0.53* | 0.46ns | 0.02ns | 0.17ns | 0.45ns | 0.57* | -0.43ns | 0.28ns |
| MTxV | 0.82** | 0.29ns | -0.39ns | -0.18ns | 0.72** | 0.68** | 0.44ns | -0.66** | 0.49* |
| MTxW | 0.54* | 0.91** | -0.95** | -0.15ns | 0.69** | 0.37ns | -0.40ns | -0.35ns | 0.16ns |
| RBD | -0.32ns | -0.83** | 0.85** | 0.07ns | -0.50* | -0.24ns | 0.74** | 0.23ns | 0.03ns |

.

* and ** indicate significant difference at 0.05 and 0.01 probability levels, respectively; ns = not significant, ND= not determined.
